# Serum uPAR as Biomarker in Breast Cancer Recurrence: A Mathematical Model

**DOI:** 10.1371/journal.pone.0153508

**Published:** 2016-04-14

**Authors:** Wenrui Hao, Avner Friedman

**Affiliations:** 1 Mathematical Biosciences Institute, The Ohio State University, Columbus, OH, United States of America; 2 Department of Mathematics, The Ohio State University, Columbus, OH, United States of America; Institute of Biochemistry and Biotechnology, TAIWAN

## Abstract

There are currently over 2.5 million breast cancer survivors in the United States and, according to the American Cancer Society, 10 to 20 percent of these women will develop recurrent breast cancer. Early detection of recurrence can avoid unnecessary radical treatment. However, self-examination or mammography screening may not discover a recurring cancer if the number of surviving cancer cells is small, while biopsy is too invasive and cannot be frequently repeated. It is therefore important to identify non-invasive biomarkers that can detect early recurrence. The present paper develops a mathematical model of cancer recurrence. The model, based on a system of partial differential equations, focuses on tissue biomarkers that include the plasminogen system. Among them, only uPAR is known to have significant correlation to its concentration in serum and could therefore be a good candidate for serum biomarker. The model includes uPAR and other associated cytokines and cells. It is assumed that the residual cancer cells that survived primary cancer therapy are concentrated in the same location within a region with a very small diameter. Model simulations establish a quantitative relation between the diameter of the growing cancer and the total uPAR mass in the cancer. This relation is used to identify uPAR as a potential serum biomarker for breast cancer recurrence.

## Introduction

Human breast cancer is a major cause of death in the United States and worldwide [1]. It is estimated that 230,000 women in the United States are diagnosed annually with invasive breast cancer, and more than 40,000 die from the disease [2]. A major factor that contributes to poor prognosis is the fact that diagnosis is often delayed due to limitation in mammography screening [3]. Poor prognosis occurs also in assessing the risk of recurrence in patients of low grade breast cancer; improving this assessment will help avoid unnecessary chemotherapy [4].

Risk factors associated with gene mutations such as BRCA1 and BRCA2, and with family history and aging have long been recognized [5]. More recent work is also looking for risk assessment that can be associated with serum biomarkers [6–8]. Three tissue biomarkers have been identified: urokinase plasminogen activator (uPA), plasminogen-activator-inhibitor (PAI-1), and tissue factor (TF) [3, 4, 9, 10]. For uPA to become active it must bind to its receptor uPAR [11]. Active uPA is extracellular matrix-degrading protease that promotes tumor progression and metastasis. It binds to plasminogen and converts it to its activated form, plasmin, a process inhibited by PAI-1 [12–16]. Plasmin mediates the activation of matrix metaloproteinase (MMP) which enables cancer cells’ migration [12, 15, 17]. TF promotes tumor by enhancing VEGF production [18]. Harbeck et. al [19] reported on an extensive 6-year study to assess the risk associated with node-negative breast cancer recurrence in terms of the levels of uPA and PAI-1. Based on this report and other studies it was concluded that tissue (uPA, PAI-1) provide predictive information about early breast cancer [4, 20]. The American Society of Clinical Oncology also recommends uPA and PAI-1 as prognostic tumor markers for breast cancer [21].

Although uPA and PAI-1 levels are elevated in breast cancer tissue, these high levels are not detected in the blood. Indeed, as reported in Rha et al.[22], the blood level of uPA and PAI-1 of the plasminogen activation system correlated with that of breast tissue in order of *R*^2^ = 0.35 for uPA and *R*^2^ = 0.11 for PAI-1. So uPA and PAI-1 are not reliable serum biomarkers. On the other hand, it was reported by Rha et al. [22], that the correlation of the level of uPAR in the blood with that of tissue is significant, with *R*^2^ = 0.61 (P = 0.001). Recently Soydine et al. [23] found that uPAR in serum and in urine of breast cancer patients (n = 180) was significantly higher than in healthy control (n = 60). Serum uPAR was also shown to be a prognostic biomarker in endometrial cancer [24].

The present paper is concerned with prognosis of breast cancer recurrence. Most commonly, recurrence occurs within 3–5 years, although the statistics of recurrence is not clear [25, 26]. When breast cancer recurs, it most often recurs in the same location as the primary cancer [27]. In the present paper we address with a mathematical model the following question: Can uPAR be used as biomarker to recognize breast cancer recurrence? We assume that after treatment of the original cancer, some cancer cells survived in the same location, occupying a small spherical region of radius *R*_0_ and that the cancer begins to grow while maintaining a spherical shape. Simulations of our mathematical model profile the cancer radius and the expression of uPAR as functions of *R*_0_ and time *t*: *R*(*R*_0_, *t*) and *uPAR*(*R*_0_, *t*). If a patient’s uPAR in tissue (surrounding the primary tumor) is measured at time *t*, say at *t* = 100 days, we can then use this measurement to determine *R*_0_ and hence also the tumor radius *R*(*R*_0_, *t*) for any time *t* after 100 days. The articles of Rha et al. [22] and Soydine et al. [23] suggest that the uPAR level in serum is siginificantly correlated and hence proportional to the level of uPAR in the tissue, hence serum uPAR could serve as a potential biomarker. When clinical data become available to more reliably confirm this proportionality coefficient, the uPAR could then actually be used as serum biomarker for breast cancer recurrence.

## Model

**Fig 1:**
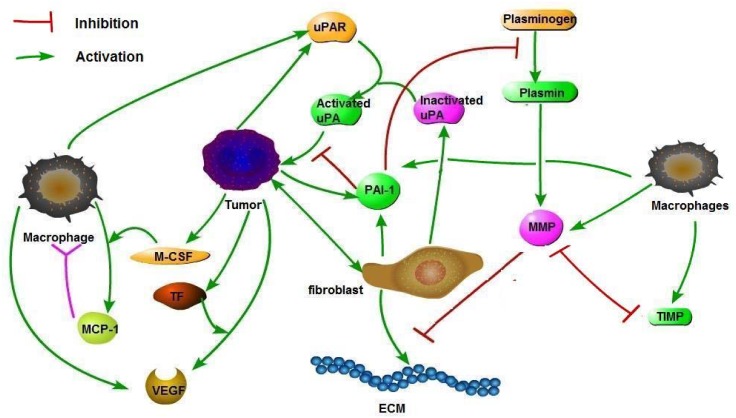
Schematic network of breast cancer with uPA, PAI-1 and uPAR: Arrows means activation; block arrow means inhibition.

The mathematical model is based on the diagram shown in Fig 1. The model includes, in addition to uPA, uPAR and PAI-1, also TF, VEGF, M-CSF, MMP and MCP-1. It also includes the cells that produce these proteins, or activated by them, namely cancer cells, fibroblasts, macrophages and endothelial cells. The variables of the model are listed in Table 1. The model is described by a system of partial differential equations (PDEs) in a radially symmetric tumor, with evolving radius *R*(*t*). The initial radius, *R*_0_ = *R*(0), is a parameter which is patient-dependent. We shall assume that the total density of cells at each point in the tissue is constant; since tumor cells proliferate, the radius *R*(*t*) is increasing with time and cells are moving with velocity **u** which is time dependent.

**Table 1:** The variables of the model; concentration and densities are in units of *g*/*cm*^3^.

*T*:	concentration of tissue factor	*V*:	concentration of VEGF
*P*:	concentration of plasmin	*u*_*PR*_:	concentration of uPAR
$u_{P}^{i}$:	concentration ofinactivated uPA	$u_{P}^{a}$:	concentration of activated uPA
*P*_*A*_:	concentration of PAI-1	*q*:	concentration of M-CSF
*p*:	concentration ofMCP-1	*M*:	macrophage density
*E*:	endothelial cell density	*T*_*β*_	TGF-*β* concentration
*G*:	EGF concentration	*f*:	fibroblast density
*C*:	cancer cell density	*w*:	concentration of oxygen
*Q*:	concentration of MMP	*Q*_*r*_:	concentration of TIMP
*ρ*:	ECM density	*R*(*t*):	radius of tumor at time *t*
**u**:	cell velocity		

### Equation for tissue factor (*T*)

The tissue factor equation is given by

$$\frac{\partial T}{\partial t}-D_{T}\Delta T\;=\;\underset{production}{\underset{︸}{A_{T}+\lambda_{TC}C}}\underset{degradation}{\underset{︸}{-d_{T}T}},$$ (1)

where the second term is the production by cancer cells [28, 29].

### Equation for VEGF (*V*)

The evolution of VEGF concentration is modeled by

$$\frac{\partial V}{\partial t}-D_{V}\Delta V\;=\;\underset{activation}{\underset{︸}{\lambda_{VC}C(1+\lambda_{VT}\frac{T}{K_{T}+T})+\lambda_{VM}M}}\underset{degradation}{\underset{︸}{-d_{V}V}}.$$ (2)

The first term on the right-hand side accounts for production of VEGF by cancer cells [28, 30, 31], a process enhanced by tissue factor [18], and the second term accounts for VEGF produced by macrophages [30, 31].

### Equation for plasmin (*P*)

Plasminogen is the inactive precursor of trypsin-like serine plasmin. When it becomes activated, it is converted to plasmin. The generation of plasmin requires the binding of uPA to plasminogen, after uPA was released from the complex uPA-uPAR and became active [32], and this binding is inhibited by PAI-1 [12–16]. For simplicity we take the concentration of plasminogen to be constant. Hence the concentration of plasmin concentration satisfies the equation

$$\frac{\partial P}{\partial t}-D_{P}\Delta P\;=\;\underset{production}{\underset{︸}{\lambda_{P}(1+\lambda_{Pu}\frac{u_{P}^{a}}{K_{P_{A}}+P_{A}})}}\underset{degradation}{\underset{︸}{-d_{P}P}}.$$ (3)

### Equation for uPAR (*u*_*PR*_)

The uPA receptor uPAR is expressed by macrophages [11, 33] and breast cancer cells [34]. Hence the equation of uPAR can be written as follows:

$$\frac{\partial u_{PR}}{\partial t}-D_{u_{PR}}\Delta u_{PR}\;=\;\underset{production}{\underset{︸}{\lambda_{u_{PR}M}M+\lambda_{u_{PR}C}C}}\underset{degradation}{\underset{︸}{-d_{u_{PR}}u_{PR}}}.$$ (4)

### Equation for uPA ($u_{P}^{i}$ and $u_{P}^{a}$)

Inactive uPA is produced by fibroblasts [35]. Hence,

$$\frac{\partial u_{P}^{i}}{\partial t}-D_{u_{P}}\Delta u_{P}^{i}\;=\;\underset{production}{\underset{︸}{\lambda_{uf}f}}\underset{degradation}{\underset{︸}{-d_{u_{P}^{i}}u_{P}^{i}}}.$$ (5)

uPA is activated when inactive uPA combines with uPAR. We take the equation for $u_{P}^{a}$ to be

$$\frac{\partial u_{P}^{a}}{\partial t}-D_{u_{P}}\Delta u_{P}^{a}\;=\;\underset{production}{\underset{︸}{\lambda_{u}u_{P}^{i}\frac{u_{PR}}{K_{u_{PR}}+u_{PR}}}}\underset{degradation}{\underset{︸}{-d_{u_{P}^{a}}u_{P}^{a}}}.$$ (6)

### Equation for PAI-1 (*P*_*A*_)

The equation of the PAI-1 concentration is given by

$$\frac{\partial P_{A}}{\partial t}-D_{P_{A}}\Delta P_{A}\;=\;\underset{production}{\underset{︸}{\lambda_{PC}C+\lambda_{Pf}f+\lambda_{PM}M}}\underset{degradation}{\underset{︸}{-d_{P_{A}}P_{A}}}.$$ (7)

The first three terms on the right-hand side account for production of PAI-1 by cancer cells [11], fibroblasts [11, 36] and macrophages [37].

### Equation for M-CSF (*q*)

The M-CSF concentration satisfies the equation

$$\frac{\partial q}{\partial t}-D_{q}\Delta q\;=\;\underset{production}{\underset{︸}{\lambda_{qC}C}}\;\underset{degradation}{\underset{︸}{-d_{q}q}},$$ (8)

where the first term of the right-hand side account for production by cancer cells [38].

### Equation for MCP-1 (*p*)

The equation of the MCP-1 concentration is given by

$$\frac{\partial p}{\partial t}-D_{p}\Delta p\;=\;\underset{production}{\underset{︸}{\lambda_{p}(w)\frac{q}{q_{0}+q}M}}\underset{degradation}{\underset{︸}{-d_{p}p}}.$$ (9)

The first term of the right-hand side accounts for production of MCP-1 by macrophages activated by M-CSF [31, 39]. Here $\lambda_{p}\left(w\right)=\lambda_{p}\frac{w}{w_{h}}$ if *w* < *w*_*h*_ and *λ*_*p*_(*w*) = *λ*_*p*_ if *w* > *w*_*h*_, where *w*_*h*_ is an appropriate hypoxic level.

### Equation for macrophages (*M*)

We assume that all cells are moving with common velocity **u**, and are subject to dispersion. The equation of macrophages density is given by

$$\frac{\partial M}{\partial t}+\nabla \cdot (uM)-D_{M}\Delta M\;=\;\underset{source}{\underset{︸}{\beta \frac{p}{K_{p}+p}M_{0}}}\underset{chemotaxis}{\underset{︸}{-\nabla (\chi_{C}M\nabla (p))}}\underset{death}{\underset{︸}{-d_{M}M}},$$ (10)

where *D*_*M*_ is the dispersion coefficient. The second term of the right-hand side accounts for chemotaxis [28, 31, 39]. Monocytes from the vascular system, with density *M*_0_, are attracted to the tissue by MCP-1, and they differentiate into macrophages. Macrophages are terminally differentiated cells. On the boundary of each blood vessel within the tumor there holds a flux condition, which we take to be

$$\frac{\partial M}{\partial n}\;-\;\tilde{\alpha_{M}}\frac{p}{K_{p}+p}M_{0}=0,\;forsome\;\tilde{\alpha_{M}}\;>\;0.$$

As in [40] we can use a homogenization method to replace these fluxes by an average source of macrophages within the tissue, and this is the first term on the right-hand side of Eq (11). We assume that the macrophages are primarily tumor-associated-macrophages (TAM) of M2 phenotype.

### Equation for endothelial cells (*E*)

Endothelial cells are chemoattracted by VEGF and their proliferation is increased by VEGF [31]. The equation for endothelial density is given by

$$\frac{\partial E}{\partial t}+\nabla \cdot (uE)-D_{E}\Delta E\;=\;\underset{chemotaxis}{\underset{︸}{-\nabla (\chi_{C}E\nabla (V))}}\underset{proliferation}{\underset{︸}{+\lambda_{E}E(1-\frac{E}{K_{E}}){\left(V-V_{0}\right)}^{+}}}\underset{death}{\underset{︸}{-d_{E}E}}.$$ (11)

where we used the notation: *X*^+^ = *X* if *X* > 0, *X*^+^ = 0 if *X* ≤ 0. The second term on the right-hand side assumes a threshold *V*_0_ below which proliferation of *E* does not occur [41, 42].

### Equation for fibroblasts (*f*)

There is a mutual enhancement in the interaction between cancer cells and fibroblasts. Cancer cells secrete TGF-*β* which increases the activation and proliferation of fibroblasts, while EGF secreted by fibroblasts increases the proliferation of cancer cells [43–50].

We write simplified equations for TGF-*β* (*T*_*β*_) and EGF(*G*):

$$\frac{dT_{\beta }}{dt}=\lambda_{T_{\beta }}C-d_{T_{\beta }}T_{\beta },\;\frac{dG}{dt}=\lambda_{G}f-d_{G}G.$$

The growth of cancer cells occurs on a time scale of days, whereas the secretion and decay of cytokines occur on a time scale of minutes to hours [51]. In order to understand the growth of cancer we simplify the model by using quasi-steady-state approximation for the equations of *T*_*β*_ and *E*, so that *T*_*β*_ = *c*_1_ *C*, *G* = *c*_2_ *f* for some constants *c*_1_ and *c*_2_. Then the fibroblasts-enhanced growth rate of fibroblast density (*f*) through *T*_*β*_ may be replaced by *λ*_*fC*_*C*, and the tumor-cell-enhanced proliferation of cancer density (*C*) through *G* may be replaced by *λ*_*Cf*_ *f*. We use the Michaelis-Menten law to express the enhanced proliferation of fibroblasts by *T*_*β*_ (i.e., by *c*_1_ *C*) because TGF-*β* activation of fibroblast and EGF enhancement of cancer cells may be limited due to the limited rate of receptors recycling associated with this process.

The equation of fibroblast density is then given by

$$\frac{\partial f}{\partial t}+\nabla \cdot (uf)-D_{f}\Delta f\;=\;\underset{proliferation}{\underset{︸}{A_{f}+\lambda_{fC}f\frac{C}{K_{C}+C}}}\underset{death}{\underset{︸}{-d_{f}f}}.$$ (12)

### Equation for cancer cells (*C*)

The equation of the cancer cells density is given by

$$\begin{array}{l} \begin{array}{lll} \frac{\partial C}{\partial t}+\nabla \cdot (uC)-D_{C}\Delta C & = & \underset{proliferation}{\underset{︸}{\left[\lambda_{C}(w)+\lambda_{Cf}\frac{f}{K_{f}+f}+\lambda_{Cu_{P}}\frac{u_{P}^{a}}{K_{P_{A}}+P_{A}}\frac{u_{PR}}{K_{u_{PR}+u_{PR}}}]\;C\;(1-\frac{C}{C_{0}}\right)}}\\ & & \underset{death}{\underset{︸}{-d_{C}C}}. \end{array}\\ \; \end{array}$$ (13)

There are three terms in the bracket: The first term is for tumor growth at rate which is oxygen dependent; the second term represents enhancement by EGF produced by fibroblasts; the third term accounts for proliferation of cancer cells by uPA as it binds to its receptor uPAR on cancer cells [11, 12, 15, 36], a process resisted by PAI-1 [14, 16]. The Michaelis-Menten law is used to represent the limited rate of receptor recycling associated with the enhancements by *f* and by *u*_*PR*_. We take $\lambda_{C}\left(w\right)=\lambda_{wC}\frac{w}{w_{h}}$ if *w* ≤ *w*_*h*_ and *λ*_*C*_(*w*) = *λ*_*wC*_ if *w* ≥ *w*_*h*_.

### Equation for oxygen (*w*)

The oxygen concentration evolves according to the equation

$$\frac{\partial w}{\partial t}-D_{w}\Delta w\;=\;\lambda_{w}E-d_{wM}wM-d_{wC}wC-d_{wf}wf.$$ (14)

The first term of the right-hand side accounts for infusion of oxygen through the blood, which is represented by the density of endothelial cells. The last three terms represent oxygen taken up by macrophages, tumor cells and fibroblasts.

### Equations for MMP (*Q*) and TIMP (*Q*_*r*_)

We have the following sets of reaction-diffusion equations for MMP and TIMP:

$$\frac{\partial Q}{\partial t}-D_{Q}\Delta Q\;=\;\underset{production}{\underset{︸}{\lambda_{QM}M(1+\lambda_{QP}\frac{P}{K_{P}+P})}}\underset{depletion}{\underset{︸}{-d_{QQ_{r}}Q_{r}Q}}\underset{degradation}{\underset{︸}{-d_{Q}Q}}$$ (15)

$$\frac{\partial Q_{r}}{\partial t}-D_{Q_{r}}\Delta Q_{r}\;=\;\underset{production}{\underset{︸}{\lambda_{Q_{r}M}M}}\underset{depletion}{\underset{︸}{-d_{Q_{r}Q}QQ_{r}}}\underset{degradation}{\underset{︸}{-d_{Q_{r}}Q_{r}}}$$ (16)

MMP and TIMP are activated by macrophages [52, 53] and MMP activation is enhanced by plasmin *P*[17], and MMP is lost by binding with TIMP, while TIMP is depleted as it blocks MMP [38, 54, 55].

### Equation for ECM (*ρ*)

Extracelluar matrix (ECM) is produced by fibroblasts [40], and is degraded by MMP [38, 54]. The equation for the density of ECM is given by

$$\frac{\partial \rho }{\partial t}+\nabla \cdot (u\rho )=\underset{production}{\underset{︸}{\lambda_{\rho f}f{\left(1-\frac{\rho }{\rho_{0}}\right)}^{+}}}\underset{degradation}{\underset{︸}{-d_{\rho }\rho -d_{\rho Q}Q\rho }},$$ (17)

### Equation for *u*

We assume that the total density of all the cells plus the density of *ρ* is constant:

M+E+f+C+ρ=const.=1. (18)

We also assume that all cells are approximately of the same volume and surface area, so that the dispersion coefficients of the all cells have the same coefficient, *D*. By adding Eqs (11)–(14) and (17), we get an equation for ∇⋅**u**:

∇⋅u+DΔρ=∑[RHS of Eqs(11),(12),(13),(14)and(17)], (19)

We can, conversely, derive Eq (18) from Eqs (11)–(14) and (19).

We assume that if a breast cancer recurs, it is because some cancer cells survived in the initial location. We also assume, to simplify the computations, that these cells are contained in a sphere of small radius *R*_0_, and that as time increases the region containing the cancer cells is a sphere with increasing radius *r* = *R*(*t*), and all the model variables are radially symmetric, that is, they are functions of (*r*, *t*) where 0 ≤ *r* ≤ *R*(*t*), *t* > 0. In particular,

$$u=u(r,t)e_{r}$$

where $e_{r}=\frac{x}{\left|x\right|}$ is the radial unit vector.

### The free boundary equation

We assume that the tumor is spherical with radius *r* = *R*(*t*), so that

$$\frac{dR}{dt}=ue_{r}=u(R(t),t),$$ (20)

where $e_{r}=\frac{x}{\left|x\right|}$ is the radial unit vector.

### Boundary conditions

We assume flux boundary conditions due to oxygen transport of the form

$$\frac{\partial X}{\partial n}+\alpha_{X}\;{\left(X\;-\;X_{0}\right)}^{+}\;=\;0$$ (21)

for *X* = *w* and *E*, where *X*_0_ is the density of oxygen or of endothelial cells within the vascular system at the boundary of the tumor. The coefficients *α*_*X*_ on the free boundary are chosen as follows: $\alpha_{w}=\tilde{\alpha_{w}}\frac{E}{K_{E}+E}$, and $\alpha_{E}=\tilde{\alpha_{E}}\frac{V}{K_{V}+V}$. We assume no-flux boundary condition for all other variables except *ρ*. Since *ρ* satisfies the hyperbolic Eq (17) with *ue*_*r*_ as the velocity of the free boundary, we do not need to prescribe a boundary condition for *ρ* on the free boundary. The radial velocity is determined from Eq (19).

### Initial conditions

We choose the following initial conditions for ECM, oxygen and cells concentration in unit of *g*/*cm*^3^: *ρ* = 0.02, *w* = *w*_*h*_, *M* = 0.07, *E* = 0.05, *f* = 0.06, and *C* = 0.4. All the cytokines are taken to be initially zero. The initial conditions do not affect the simulation results after a few weeks. However, as will be shown, the very small value of *R*(0) plays a critical role in the tumor growth profile.

## Results

**Fig 2:**
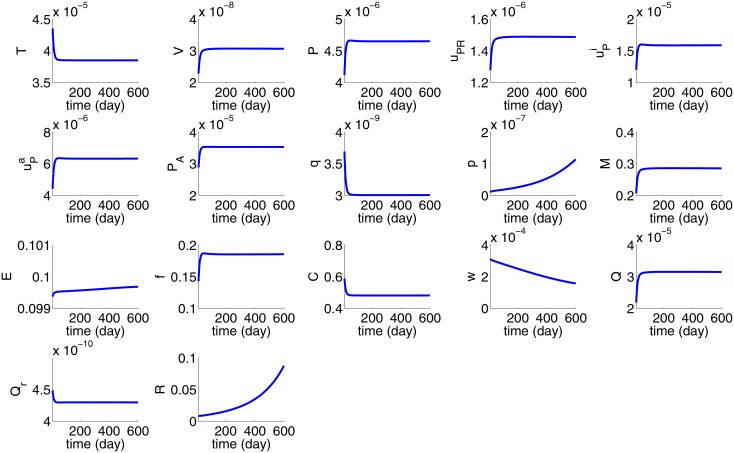
Average concentration of cytokines, average density of cells, and tumor radius *R*(*t*) for the first 600 days with *R*(0) = 10^−2^ cm = 100 *μm*. All the parameters are as in Tables 2 and 3.

**Fig 3:**
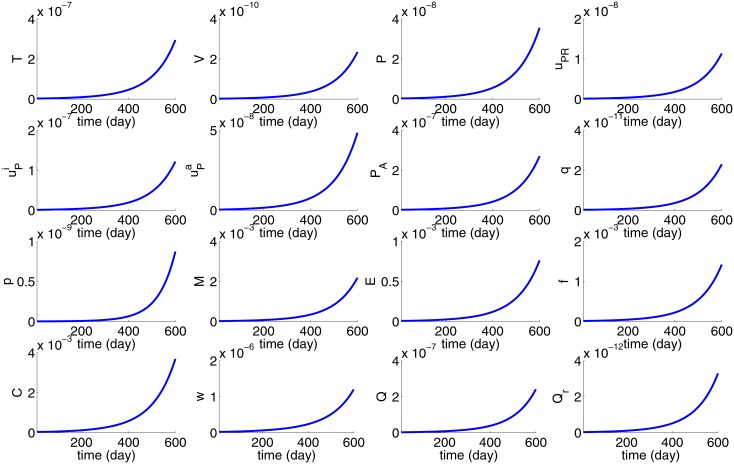
The total mass of cells and cytokines for the first 600 days with *R*(0) = 10^−2^ cm = 100 *μm*. All the parameters are as in Tables 2 and 3.

In Figs 2 and 3 we take *R*(0) = 10^−2^ cm = 100*μm*; at this initial size the tumor may contain a few thousand cells, including cancer cells. We define the average density/concentration (*Ave*) and total mass (*TM*) as follows

$$Ave=\frac{1}{R^{3}(t)}\int_{0}^{R(t)}X_{i}r^{2}dr,TM=4\pi \int_{0}^{R(t)}X_{i}r^{2}dr.$$

Fig 2 shows the profiles of the average density of cells and concentration of proteins for the first 600 days, as the tumor radius *R*(*t*) continues to grow, while Fig 3 shows the total mass of cells and proteins. Although the total mass of all the variables is growing continuously, the averages are not all monotone increasing. Cancer density dips for the first 80 days probably due to hypoxic conditions as the density of endothelial cells is still small (VEGF is still small). The average of oxygen is decreasing as cancer cells are increasing and consuming more oxygen, while the consumption of oxygen by macrophages, fibroblasts, and epithelial cells, combined, is also slightly increasing. uPA is growing after 80 days when fibroblast density begins to increase; PAI-1 mimics the profile of active uPA, while the concentrations of uPAR is relatively stable.

In estimating some of the parameters we assumed steady state of averages of densities of cells and concentrations of proteins, except for *p* and *w*. Fig 2 shows agreement with these assumed steady state averages. Thus the model simulations in Fig 2 are consistent with the assumptions made in the parameters estimation.

Harbeck et al. [20] measured the concentrations of uPA and PAI-1 in breast cancer survivors by the protein nanogram of protein antigen per milligram of tissue protein of breast tissue. They determined that when the risk of recurrence is high, PAI-1≈5×uPA. This proportion between PAI-1 and active uPA is seen also in Fig 2.

For the purpose of developing serum biomarkers we are interested in the total mass of uPAR in the growing tumor, rather than in the average density. Fig 3 shows that the total mass of cells and cytokines are increasing in time, but at different rates.

So far we have taken *R*(0) fixed at *R*_0_ = 10^−2^ cm. We next want to use the model for diagnostic purposes. Our goal is to determine from one measurement of the total mass of uPAR at time *t*_0_ after the primary breast cancer treatment the radius of the tumor at the same time *t*_0_ and at any subsequent time *t*_1_, *t*_1_ > *t*_0_. To do this we take *R*_0_ ∈ (10^−2^, 5 × 10^−2^) cm as a potential initial radius. For each *R*_0_, we compute the tumor radius at time *t*, *R* = *R*(*R*_0_, *t*) and the total mass of uPAR at time *t*, uPAR(*R*_0_, *t*).

**Fig 4:**
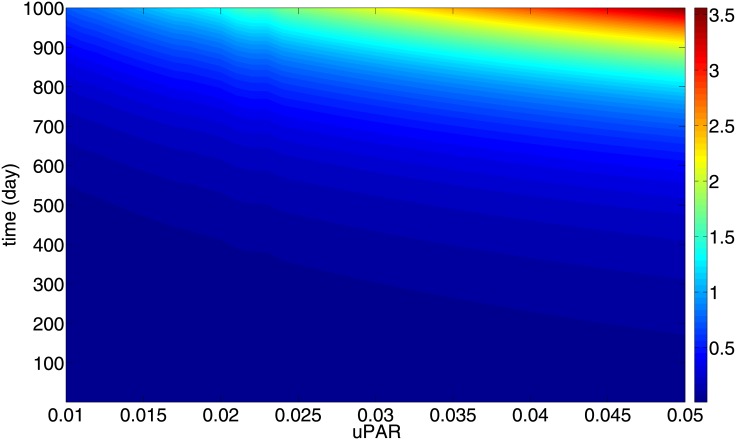
Color map for *R*(*t*). *R*_0_ ranges from 0.01 to 0.05 cm and *t* ranges from *t* = 0 to *t* = 1000 days. Color represents the size of the radius of the cancer. All the parameters are as in Tables 2 and 3.

**Fig 5:**
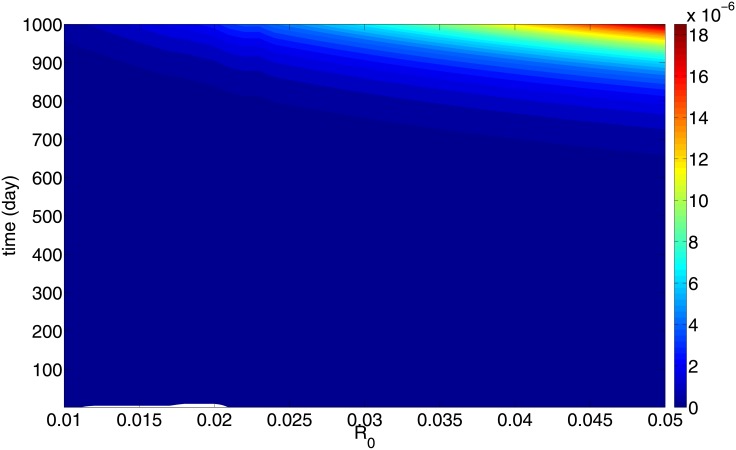
Color map for the total mass of uPAR. *R*_0_ ranges from 0.01 to 0.05 cm and *t* ranges from *t* = 0 to *t* = 1000 days. Color represents the total mass of uPAR. All the parameters are as in Tables 2 and 3.

**Fig 6:**
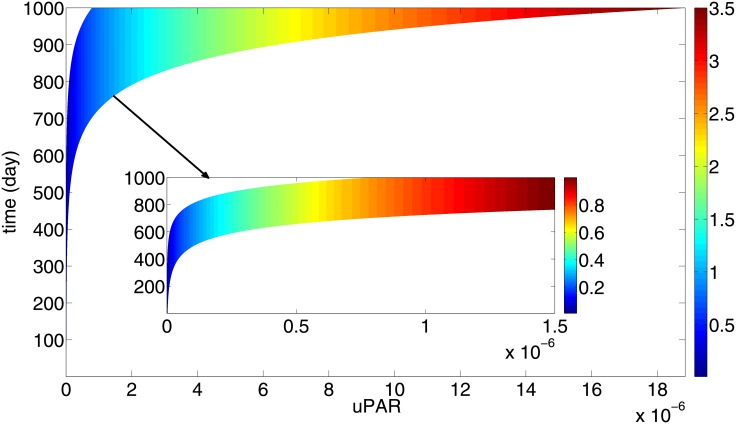
Color map for the total mass of uPAR(t) v.s. R(t). For any time *t*, 0 < *t* < 1000 days, measurement uPAR in *gm*/*cm*^3^ (on the horizontal axis) determines the size of the radius of the cancer in cm, using the column color. All the parameters are as in Tables 2 and 3.

Fig 4 shows *R* and Fig 5 shows the total mass of uPAR, at any (*R*_0_, *t*) in the range *R*_0_ ∈ (10^−2^, 5 × 10^−2^) cm and 0 < *t* < 1000 days. From these two figures we can generate a mapping from total uPAR(t) to R(t) which is independent of *R*_0_, as follows: From a value of uPAR at some time *t* after primary breast cancer treatment, we estimate, by using Fig 4, the corresponding parameter *R*_0_. We can then use Fig 5 to predict the value of R corresponding to this *R*_0_ and the time *t*. The mapping is uPAR(t)→R(t) is shown in Fig 6, where the color bar determines the value of *R*(*t*) for a given pair of uPAR and *t*.

The color map in Fig 6 is a prognostic map for recurrent breast cancer: when a patient’s uPAR is measured *t* days after the primary breast cancer treatment, the color bar in Fig 6 predicts the size of the recurrent tumor.

## Discussion

There are several mathematical models of breast cancer focusing on different aspects of the disease [28, 38, 56–58]. Our model is the first one to focus on biomarkers associated with the risk of breast cancer recurrence.

Cancer recurrence occurs in 10 to 20 percent of all breast cancer survivors. In order to avoid unnecessary radical treatment, it is important to diagnose a recurrent cancer as early as possible. Although several tissue biomarkers have been identified, biopsy cannot be frequently repeated. This motivated the present study of focusing on potential serum biomarkers which are non-invasive. Of all the plasmiogen system tissue biomarkers only uPAR concentration significantly correlates with uPAR concentration in blood [22, 23]. For this reason we developed in this paper a mathematical model that quantifies the relation between tissue uPAR and the size of a recurrent cancer (Fig 6).

The mathematical model is represented by a system of partial differential equations in a growing tumor with radius *R*(*t*). We assume a very small initial radius *R*_0_ ∈ (10^−2^, 5 × 10^−2^) cm, corresponding to cancer cells that survived after primary breast cancer treatment. The radius *R*_0_ may vary from one patient to another. Nevertheless, Fig 6 shows that a patient’s uPAR measured at any time within 1000 days after primary breast cancer treatment, can be used to estimate the cancer size (i.e., its radius) by the color bar. Furthermore, the initial *R*_0_ can then be determined from Fig 4, and hence, by Fig 5 also the radius *R*(*t*) for all subsequent times.

While the measurement of serum biomarkers for patient survival after primary breast cancer treatment is still being debated [8], we propose here uPAR as a potential serum biomarker. When clinical data become available to enable us to estimate the precise proportion of uPAR tissue concentration to plasma concentration, uPAR could then be used as plasma biomarker which informs the size of a recurrent tumor. The simulation results of Figs 4–6 can be extended to include smaller values of *R*_0_, e.g. 0.005 ≤ *R*_0_ ≤ 0.01. For such values, it takes a significantly longer time for the tumor radius to reach a size that can be detected by self examination.

The simulation results rely heavily on parameters taken from the literature, sometimes in a different context, and on parameters estimated in this paper, sometimes rather crudely. For this reason we performed sensitivity analysis to determine how the radius of the tumor will vary when some of the parameters are increased or decreased.

In developing the mathematical model we made several simplifying assumptions: (i) the tumor is radially symmetric; (ii) the total density of cells at each point of the tissue is constant; and (iii) the cells are approximately of the same volume and surface area making the dispersion coefficient of cells equal. Furthermore, the mathematical model is minimal in the sense that includes just the plasminogen system (of uPA, uPAR, PAI-1), plasmin and MMP, tissue factor, VEGF, MCP-1, M-CSF, and the cells that produce these proteins or are activated by them, namely, macrophages, endothelial cells, fibroblasts and cancer cells. For these reasons, this work should be viewed as providing just a first step upon which a more elaborate and more comprehensive model could be developed in the future. When new data of uPAR expression level in patients of breast cancer recurrence become available, some of the parameters of the model will accordingly be adjusted to make the model simulations agree with patients data. Additional cytokines may then also be included, and the assumption of a spherical tumor may be changed to better reflects tumor histology.

## Materials and Methods

All the parameters of the model are listed in Tables 2 and 3. Some of them are taken from the literature, while all the rest are estimated in this section.

**Table 2:** Parameters’ description and value.

Parameter	Description	Value
*D*_*T*_	diffusion coefficient of tissue factor	0.111 *cm*^2^day^−1^ estimated
*D*_*V*_	diffusion coefficient of VEGF	8.64 × 10^−2^ *cm*^2^ day^−1^[59]
*D*_*P*_	diffusion coefficient of plasmin	0.212 *cm*^2^day^−1^ estimated
*D*_*u*_*PR*__	diffusion coefficient for uPAR	8.64 × 10^−7^ *cm*^2^ day^−1^[54]
*D*_*u*_*p*__	diffusion coefficient of uPA	0.117 *cm*^2^day^−1^ estimated
*D*_*P*_*A*__	diffusion coefficient of PAI-1	0.127 *cm*^2^day^−1^ estimated
*D*_*q*_	diffusion coefficient of M-CSF	0.013 *cm*^2^day^−1^[54]
*D*_*p*_	diffusion coefficient for MCP-1	1.29 × 10^−2^ *cm*^2^ day^−1^[54]
*D*_*M*_	diffusion coefficient of macrophages	8.64 × 10^−7^ *cm*^2^ day^−1^[54]
*D*_*E*_	diffusion coefficient for endothelial cells	8.64 × 10^−7^ *cm*^2^ day^−1^[54]
*D*_*f*_	diffusion coefficient forfibroblasts	8.64 × 10^−7^ *cm*^2^ day^−1^[54]
*D*_*C*_	diffusion coefficient for cancer cells	8.64 × 10^−7^ *cm*^2^ day^−1^[54]
*D*_*w*_	diffusion coefficient for oxygen	4.32 × 10^−2^ *cm*^2^ day^−1^[54]
*D*_*Q*_	diffusion coefficient of MMP	4.32 × 10^−2^ *cm*^2^ day^−1^[54]
*D*_*Q*_*r*__	diffusion coefficient for TIMP	4.32 × 10^−2^ *cm*^2^ day^−1^[54]
*A*_*T*_	production rate of tissue factor	3.23 × 10^−5^ g/ml/day estimated
*λ*_*TC*_	production rate of tissue factor by cancer cell	5.7 × 10^−5^ day^−1^ estimated
*λ*_*VC*_	production rate of VEGF by cancer cell	2 × 10^−8^ *g*/*ml*/day estimated
*λ*_*VT*_	production rate of VEGF by TF	2 estimated
*λ*_*VM*_	production rate of VEGF by macrophages	2 × 10^−6^ *g*/*ml*/day estimated
*λ*_*P*_	activation rate of plasmin	2.42 × 10^−6^ g/ml/day estimated
*λ*_*Pu*_	activation rate of plasmin by uPA	10.5 estimated
*λ*_*u*_*PR*_*M*_	production rate of uPAR by macrophages	6.21 × 10^−6^ day^−1^ estimated
*λ*_*u*_*PR*_*C*_	production rate of uPAR by cancer	1.242 × 10^−6^ day^−1^ estimated
*λ*_*uf*_	production rate of uPA by fibroblasts	2.057 × 10^−4^ day^−1^ estimated
*λ*_*u*_	production rate of uPA	1.92 day^−1^ estimated
*λ*_*PC*_	activation rate of PAI-1 by cancer cells	8 × 10^−5^ day^−1^ estimated
*λ*_*Pf*_	activation rate of PAI-1 by fibroblasts	8.4 × 10^−4^ day^−1^ estimated
*λ*_*PM*_	activation rate of PAI-1 by macrophages	4 × 10^−4^ day^−1^ estimated
*λ*_*qC*_	production rate of GM-CSF by cancer cell	3 × 10^−8^ day^−1^[38]
*λ*_*p*_	production rate of MCP-1 by macrophages	1.9 × 10^−6^ day^−1^[38]
*λ*_*E*_	production rate of endothelial cells	0.7 day^−1^[31]
*A*_*f*_	based production rate of fibroblasts	10^−3^ *g*/*ml*/day [40]
*λ*_*fC*_	production rate of fibroblasts	5 × 10^−3^ day^−1^ estimated
*β*	flux rate of monocytes	0.3 day^−1^ estimated
*λ*_*Cf*_	production rate of cancer cells	0.06 day^−1^ estimated
*λ*_*Cu*_*P*__	production rate of cancer cell by uPA	0.05 day^−1^ estimated
*λ*_*wC*_	production rate of cancer by oxygen	0.6 g/cm^3^day^−1^ estimated
*λ*_*w*_	production rate of oxygen by endothelial cells	7 × 10^−2^[31, 38]
*λ*_*QM*_	production rate of MMP by macrophages	3 × 10^−4^ day^−1^[40]
*λ*_*QP*_	production rate of MMP by plasmin	2 estimated
*λ*_*Q*_*r*_*M*_	production rate of TIMP by macrophages	6 × 10^−5^ day^−1^[40]
*λ*_*ρf*_	activation rate of ECM by fibroblasts	3 × 10^−3^ day^−1^[40]

**Table 3:** Parameters’ description and value.

Parameter	Description	Value
*d*_*T*_	degradation rate oftissue factor	1.85 day^−1^[64]
*d*_*V*_	degradation rate of VEGF	12.6 day^−1^[31]
*d*_*P*_	degradation rate of plasmin	1.39 day^−1^[67]
*d*_*u*_*PR*__	degradation rate of uPAR	1.38 day^−1^[76, 77]
$d_{u_{P}^{a}}$	degradation rate of active uPA	3.2 day^−1^[76, 77]
$d_{u_{P}^{i}}$	degradation rate of inactive uPA	2.4 day^−1^[76, 77]
*d*_*P*_*A*__	degradation rate of PAI-1	8.32 day^−1^[80]
*d*_*q*_	degradation rate of GM-CSF	4.8 day^−1^[38]
*d*_*p*_	degradation rate of MCP-1	1.73 day^−1^[31, 54]
*d*_*M*_	death rate of macrophages	0.015 day^−1^[54]
*d*_*E*_	degradation rate of endothelial cells	0.69 day^−1^[54]
*d*_*f*_	death rate of fibroblasts	1.66 × 10^−2^ day^−1^[40]
*d*_*C*_	death rate of cancer cells	0.5 day^−1^[31]
*d*_*wM*_	consumption rate of oxygen by macrophages	80 *ml*/*g*/day [31]
*d*_*wC*_	consumption rate of oxygen by cancer cells	40 *ml*/*g*/day [31]
*d*_*wf*_	consumption rate of oxygen by fibroblasts	80 *ml*/*g*/day estimated
*d*_*QQ*_*r*__	binding rate of MMP to TIMP	4.98 × 10^8^ *cm*^3^ *g*^−1^ day^−1^[40, 54]
*d*_*Q*_	degradation rate of MMP	4.32 day^−1^[40, 54]
*d*_*Q*_*r*_*Q*_	binding rate of TIMP to MMP	1.04 × 10^9^ *cm*^3^ *g*^−1^ day^−1^[40, 54]
*d*_*Q*_*r*__	degradation rate of TIMP	21.6 day^−1^[40, 54]
*d*_*ρ*_	based degradation rate of ECM	0.37 day^−1^[40, 54]
*d*_*ρQ*_	degradation rate of ECM by MMP	2.59 × 10^7^ *cm*^3^ *g*^−1^ day^−1^[40, 54]
*K*_*T*_	TF half-saturation	10^−4^ *gcm*^−3^ estimated
*K*_*P*_*A*__	PAI-1 half-saturation	4.19 × 10^−6^ g/ml estimated
*K*_*u*_*PR*__	uPAR half-saturation	1.8 × 10^−6^ g/ml estimated
*K*_*p*_	MCP-1 half-saturation	2 × 10^−7^ g/ml estimated
*K*_*E*_	carrying capacity of endothelial cells	5 × 10^−3^ *gcm*^−3^[31]
*K*_*C*_	cancer cells half-saturation	0.5 *gcm*^−3^ estimated
*K*_*f*_	fibroblast half-saturation	0.1 *gcm*^−3^ estimated
*K*_*P*_	plasmin half-saturation	4.4 × 10^−6^ g/ml estimated
*K*_*V*_	VEGF half-saturation	7 × 10^−8^ g/ml estimated
*M*_0_	monocytes density in the blood	5 × 10^−5^ g/ml [54]
*V*_0_	threshold VEGF concentration	3.65 × 10^−10^ *gcm*^−3^[31]
*C*_0_	carrying capacity of cancer cells	0.75 *gcm*^−3^[31]
*q*_0_	GM-CSF half saturation	10^−9^ *gcm*^−3^[38]
*w*_0_	oxygen saturation	4.65 × 10^−4^ g/ml [31]
*E*_0_	endothelial cells density at tumor microenviroment	2.5 × 10^−3^ g/ml [31]
*ρ*_0_	ECM saturation	10^−3^ g/ml [54]
*χ*_*C*_	chemotactic coefficient	10 [54]
*w*_*h*_	oxygen half-saturation	10^−4^ *gcm*^−3^[31]
$\tilde{\alpha_{w}}$	influx rae for oxygen	1 estimated
$\tilde{\alpha_{E}}$	influx rate for endothelial cells	1 estimated

### Diffusion coefficients

The diffusion coefficients of proteins (*Y*) are proportional to the molecular surface area [54], which is proportional to $M_{Y}^{2/3}$, where *M*_*Y*_ is the molecular weight [54]. Accordingly, we can have the following relation:

$$D_{Y}=\frac{M_{Y}^{2/3}}{M_{V}^{2/3}}D_{V},$$

where *M*_*V*_ and *D*_*V*_ are the molecular weight and diffusion coefficient of VEGF. Since *D*_*V*_ = 8.64 × 10^−2^ *cm*^2^ day^−1^[59], *M*_*V*_ = 24 kDa [60] and *M*_*P*_ = 92 kDa [61], *M*_*u*_*P*__ = 38 kDa [56], *M*_*T*_ = 35 kDa [62] and *M*_*P*_*A*__ = 43 kDa [63], we get *D*_*P*_ = 0.212 *cm*^2^day^−1^, *D*_*u*_*P*__ = 0.117 *cm*^2^day^−1^, *D*_*T*_ = 0.111 *cm*^2^day^−1^ and *D*_*P*_*A*__ = 0.127 *cm*^2^day^−1^.

### Eq (1)

- *d*_*T*_: The tissue factor half life is 9 hours [64], hence *d*_*T*_ = 1.85 day^−1^.
- *A*_*T*_: The concentration of tissue factor in cancer is 3.5 × 10^−5^ g/ml [65]. The ratio of tissue factor in healthy to disease in plasma is 1:2 [66]. We assume larger ratio in breast tissue, so that in the healthy case *T* = 2 × 10^−4^ g/ml and take *K*_*T*_ = 10^−4^ g/ml. From Eq (1), in steady state for the healthy case, we get *A*_*T*_ = *d*_*T*_ *T* = 3.23 × 10^−5^ g/ml/day.
- *λ*_*TC*_: We assume that most of the tumor is populated with cancer cells, and take *C* = 0.5 g/ml. As in the deviation of Eq (12), to simplify the model and to estimate some of the parameters, we assume that cytokine equations are in steady-state. From the steady-state of Eq (1) in disease state we have *A*_*T*_ + *λ*_*C*_ *C* = *d*_*T*_ × 3.5 × 10^−5^, so that *λ*_*TC*_ = 5.7 × 10^−5^ day^−1^.

### Eq (2)

According to (31, 39) *λ*_*VC*_ varies in the range of 10^−21^ to 10^−20^ in units of g/s/cell, and the production of VEGF by tumor associated macrophages is far larger than the production of VEGF by cancer cells. Accordingly we take *λ*_*VC*_ = 2 × 10^−8^/day and *λ*_*VM*_ = 2 × 10^−6^/day. We also assume that T enhances cancer-cells production by less than 200% and take *λ*_*VT*_ = 2.

### Eq (3)

- *d*_*P*_: The half life of plasmin is 0.5 day [67], hence *d*_*P*_ = 1.39/day.
- *λ*_*Pu*_: uPAR in disease is 3/2 uPAR in healthy case [68]. Plasminogen binding to cancer cells was increased by 3/2 in disease compared to the healthy [69]. Hence we assume that
  $$\lambda_{Pu}\frac{u_{P}^{a}}{K_{P_{A}}+P_{A}}=\frac{3}{2},$$
  where *P*_*A*_ = 8.39 × 10^−6^[70], and $u_{P}^{a}=1.8\times 10^{-6}$ g/ml [70, 71]. By taking $K_{P_{A}}=\frac{1}{2}P_{A}=4.19\times 10^{-6}$, we get *λ*_*Pu*_ = 10.5.
- *λ*_*P*_: The molecular weight of plasminogen is 92 kDa [61] while molecular weight of uPA is 38 kDa [56]. The concentration of uPA in breast cancer is 1.8 × 10^−6^ g/ml [70, 71]. We assume that the number of uPA proteins in plasma is the same as the number of plasmin proteins, so that
  $$\frac{\text{the concentration of plasmin}}{\text{the concentration of uPA}}=\frac{\text{92 kDa}}{\text{38 kDa}}.$$
  Therefore, the concentration of plasmin in breast cancer is approximately 4.4 × 10^−6^ g/ml. By Eq (3), we have
  $$\lambda_{P}(1+3/2)=d_{P}P,$$
  which implies *λ*_*P*_ = 2.42 × 10^−6^ g/ml/day.

### Eq (4)

- *d*_*u*_*PR*__: The half life of uPAR is 12 hours [72], therefore *d*_*u*_*PR*__ = 1.38/day.
- *λ*_*u*_*PR*_*M*_ and *λ*_*u*_*PR*_*C*_: The concentration of uPAR in breast cancer is 1.8 × 10^−6^ g/ml [70, 71]. The concentration of uPAR in normal healthy breast is significantly smaller [73]; we take it to be approximately 10 times smaller. In healthy case, *M* = 0.04 g/ml [74], and from
  $$\lambda_{u_{PR}M}M-d_{u_{PR}}u_{PR}=0$$
  we get, *λ*_*u*_*PR*_*M*_ = 6.21 × 10^−6^ day^−1^.
  In disease case, *M* = 0.3 g/ml [75]. Taking *C* = 0.5 g/ml in the steady state equation
  $$\lambda_{u_{PR}M}M+\lambda_{u_{PR}C}C-d_{u_{PR}}u_{PR}=0,$$
  we get *λ*_*u*_*PR*_*C*_ = 1.242 × 10^−6^ day^−1^.

### Eqs (5) and (6)

- $d_{u_{P}^{i}}$ and $d_{u_{P}^{a}}$: The half life of activated uPA is 5 hours [76, 77], therefore $d_{u_{P}^{a}}=3.2$/day. We assume that inactivated uPA degrades slower, and take $d_{u_{P}^{i}}=2.4$/day.
- *λ*_*uf*_: The concentration of uPA in normal healthy tissue is estimated to $u_{P}^{i}=6\times 10^{-6}$ g/ml [78], and fibroblast density is 0.07 g/ml [40]. From
  $$\lambda_{uf}f-d_{u_{P}^{i}}u_{P}^{i}=0$$
  we get *λ*_*uf*_ = 2.057 × 10^−4^ day^−1^.
- *λ*_*u*_: We use the steady state equation
  $$\lambda_{u}u_{P}^{i}\frac{u_{PR}}{K_{u_{PR}}+u_{PR}}-d_{u_{P}^{a}}u_{P}^{a}=0$$
  where we take *K*_*u*_*PR*__ = *u*_*PR*_ = 1.8 × 10^−6^ g/ml, and $u_{p}^{a}=1.8\times 10^{-6}$ g/ml [70, 71]. Since $u_{P}^{i}/u_{P}^{a}$ = 3.3, we get *λ*_*u*_ = 1.92 day^−1^.

### Eq (7)

The PAI-1 concentration in breast cancer is 12 ng/mg [70] (and $\frac{\text{mg}\;\text{protein}}{\text{mg}}=1.43$ [79]), which implies that *P*_*A*_ = 12 × 10^−9^/1.43/10^−3^ = 8.39 × 10^−6^ g/ml. Since *d*_*P*_*A*__ = 8.32 day^−1^[80], we have *d*_*P*_*A*__ *P*_*A*_ = 7 × 10^−5^ g/ml/day. In the steady state, we have

$$\lambda_{PC}C+\lambda_{Pf}f+\lambda_{PM}M=d_{P_{A}}P_{A}=7\times 10^{-5}g/ml/\text{day}.$$

However, since the tumor is growing, the left-hand side should be larger than 7 × 10^−5^. We take the left-hand side to be 4 times larger than 7 × 10^−5^. We also assume that the first term *λ*_*PC*_ *C* contributes $\frac{1}{7}$-th, and that, the remaining two terms contribute each $\frac{3}{7}$-th of the total 7 × 10^−5^. From *λ*_*PC*_ *C* = 4 × 10^−5^ g/ml/day, where *C* = 0.5 g/ml, we get *λ*_*PC*_ = 8 × 10^−5^/day. Similarly, from *λ*_*Pf*_ *f* = *λ*_*PM*_ *M* = 1.2 × 10^−4^ g/ml/day and *f* = 0.14 g/ml, *M* = 0.3 g/ml, we get *λ*_*Pf*_ = 8.4 × 10^−4^/day and *λ*_*PM*_ = 4 × 10^−4^/day.

### Eq (10)

From steady state of Eq (9) with *M* = 0.3 *g*/*cm*^3^, *λ*_*p*_(*w*) ∼ *λ*_*p*_, *d*_*p*_ = 1.73/day and *q*/(*q*_0_ + *q*) ∼ 1/2, we take an approximate steady state of *p* to be 2 × 10^−7^ *g*/*cm*^3^, and *K*_*p*_ = 2 × 10^−7^ *g*/*cm*^3^. The parameter *β* is unknown; in [40] it was chosen to be 0.2/day; here we take it to be 0.3/day.

### Eq (12)

In Eq (12) production term $\lambda_{fC}f\frac{C}{K_{C}+C}$ is due to cytokines secreted by cancer cells. We assume that this term is only a fraction of the death rate *d*_*f*_ *f* of fibroblasts, where *d*_*f*_ = 16.6 × 10^−3^/day, and take *λ*_*fC*_ = 5 × 10^−3^/day.

### Eq (13)

*λ*_*C*_(*w*) accounts for the proliferation rate minus the death rate by necrosis, while *d*_*C*_ is the death rate by apoptosis. In transgenic mice *λ*_*C*_(*w*) is large [31, 38], and cancer develops within a few days. Since breast tumor in human develops much slower, on the time scale of years, we take $\lambda_{C}\left(w\right)=0.6\frac{w}{w_{h}}$ day^−1^ if *w* < *w*_*h*_ and *λ*_*C*_(*w*) = 0.6 day^−1^ if *w* > *w*_*h*_, while *d*_*C*_ = 0.5 day^−1^[31]. We assume that the enhanced proliferation rate by fibroblast and by $u_{P}^{a}$ binding to *u*_*PR*_ are small, and take *λ*_*Cf*_ = 0.06 day^−1^ and *λ*_*Cup*_ = 0.05 day^−1^.

In steady healthy state, we have by Eq (12), *A*_*f*_ = *d*_*f*_ *f* where *A*_*f*_ = 10^−3^ *g*/*cm*^3^/day, *d*_*f*_ = 1.66 × 10^−2^/day. So *f* = 6.02 × 10^−2^ *g*/*cm*^3^. Accordingly, we take the half-saturation *K*_*f*_ = 0.1 *g*/*cm*^3^

### Eq (14)

We assume that fibroblasts consume oxygen at the same rate as macrophages, so that *d*_*wf*_ = *d*_*wM*_ = 80 *cm*^3^/*g*/day by [31].

### Eq (15)

From the steady state of Eq (3), with no activated uPA, we get $P=\frac{\lambda_{P}}{d_{P}}$. Since *d*_*P*_ = 1.39/day [67] and *λ*_*P*_ was estimated (in Eq (3)) by 2.42 × 10^−6^ *g*/*cm*^3^/day, we get *P* = 1.74 × 10^−6^ *g*/*cm*^3^. With active uPA in Eq (3), this value of *P* should be increased by the factor $1+\lambda_{P_{u}}\frac{u_{P}^{a}}{K_{P_{A}}+P_{A}}$, so accordingly we take the half-saturation *K*_*P*_ to be 4.4 × 10^−6^ *g*/*cm*^3^.

### Boundary conditions

Since most VEGF is produced by tumor associated macrophages [31, 39]. the steady state of Eq (2) yields

$$(1+\varepsilon )\lambda_{VM}M=d_{V}V$$

where *ε* < 1, *d*_*V*_ = 12.6/day [31] and *λ*_*VM*_ = 2 × 10^−6^/day. Taking *M* = 0.3 g/ml and *ε* = 1/2, we get the approximate value *V* = 7 × 10^−8^ g/ml. We accordingly take *K*_*V*_ = 7 × 10^−8^. We also take $\tilde{\alpha_{w}}$ and $\tilde{\alpha_{E}}$ to be of order 1, and for simplicity choose $\tilde{\alpha_{w}}=\tilde{\alpha_{E}}=1$; however, other choices affect the model simulations only very little (not shown here).

### Sensitivity analysis

**Fig 7:**
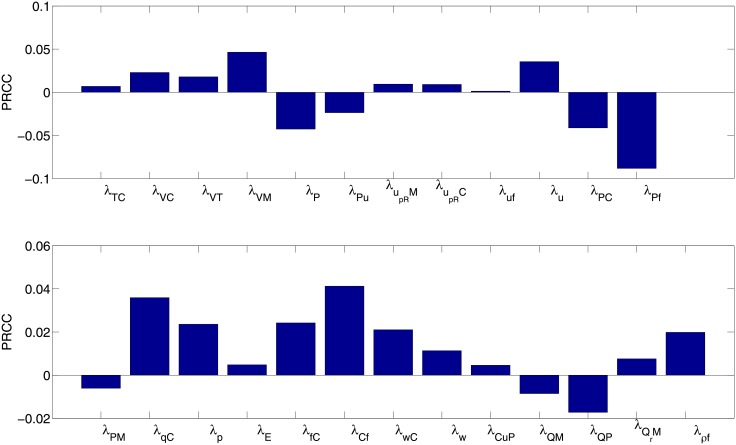
The sensitivity analysis for the cytokine production rates. The figure shows the partial rank correlation (PRCC) between the cytokine production rate and the radius of tumor. All the parameters are as in Tables 2 and 3.

We performed sensitivity analysis on the all production parameters of the system (1)-(17). Following the method in [81], we performed Latin hypercube sampling and generated 1000 samples to calculate the partial rank correlation (PRCC) and the p-values with respect to the radius of the tumor at day 600. The results are shown in Fig 7 (The p-value was <0.01).

The most positively correlated production parameters are *λ*_*VM*_ (the production of VEGF by macrophages), *λ*_*u*_ (the production uPA activator), *λ*_*Cf*_ (*λ*_*Cf*_ and *λ*_*fC*_ represent the cross-talk between cancer cells and fibroblasts, which increases the number of cancer cells). The most negatively correlated production parameters are *λ*_*Pf*_ and *λ*_*PC*_ (which increase, together with *λ*_*PM*_, the production of PAI-1, thus increasing the blockage on uPA and the consequently proliferation of cancer cells), and *λ*_*P*_ (which increases plasmin, and hence also PAI-1).

The remaining parameters are mildly correlated to tumor growth, and their correlation (+ or -) is agreement with the model description in Fig 1.

## Computational Method

In order to illustrate our numerical method, we consider the following convection-diffusion equation:

$$\frac{\partial X}{\partial t}+div(vX)=D\nabla^{2}X+F_{X},$$ (22)

where *F*_*X*_ accounts for all the ‘active’ terms. Since the model we consider is a free boundary problem, we employ the moving mesh method to compute it. We write Eq (33) can be written in the total derivative form

$$\frac{dX(r(t),t)}{dt}+div(v)X=D\nabla^{2}X(r(t),t)+F_{X}.$$

Let $r_{i}^{n}$, $X_{i}^{n}$ denote numerical approximations of i-th grid point and $X(r_{i}^{n},t)$, respectively, when *t* = *nτ*, where *τ* is the time stepsize. The discretization is derived by the explicit Euler finite difference scheme, i.e.,

$$\frac{X_{i}^{n+1}-X_{i}^{n}}{\tau }+(\frac{v_{r}}{r_{i}^{n}}+v_{i}^{n})X_{i}^{n}=D(X_{rr}+\frac{X_{r}}{r_{i}^{n}})+F_{X},$$

where $X_{r}=\frac{h_{-1}^{2}X_{i+1}^{n}-h_{1}^{2}X_{i-1}^{n}-\left(h_{1}^{2}-h_{-1}^{2}\right)X_{i}^{n}}{h_{1}\left(h_{-1}^{2}-h_{1}h_{-1}\right)}$, $X_{rr}=2\frac{h_{-1}X_{i+1}^{n}-h_{1}X_{i-1}^{n}+\left(h_{1}-h_{-1}\right)X_{i}^{n}}{h_{1}\left(h_{1}h_{-1}-h_{-1}^{2}\right)}$, and $h_{-1}=r_{i-1}^{n}-r_{i}^{n}$, $h_{1}=r_{i+1}^{n}-r_{i}^{n}$. Then the mesh is moving by $r_{i}^{n+1}=r_{i}^{n}+v_{i}^{n}\tau$, where $v_{i}^{n}$ is solved by the velocity equation. In order to make the Euler method stable, we take $\tau \leq \frac{min\{h_{1},h_{-1}\}}{2D}$.
